# Neurogenesis in the dentate gyrus: carrying the message or dictating the tone

**DOI:** 10.3389/fnins.2013.00050

**Published:** 2013-04-04

**Authors:** Verónica C. Piatti, Laura A. Ewell, Jill K. Leutgeb

**Affiliations:** Neurobiology Section, Division of Biological Sciences, Center for Neural Circuits and Behavior, University of CaliforniaSan Diego, La Jolla, CA, USA

**Keywords:** dentate gyrus, neurogenesis, sparse coding, inhibition, pattern separation

## Abstract

The dentate gyru*s* (DG) is a region in the mammalian brain critical for memory encoding with a neuronal architecture and function that deviates considerably from other cortical areas. One of the major differences of the DG compared to other brain regions is the finding that the dentate gyrus generates new principal neurons that are continuously integrated into a fully functional neural circuit throughout life. Another distinguishing characteristic of the dentate network is that the majority of principal neurons are held under strong inhibition and rarely fire action potentials. These two findings raise the question why a predominantly silent network would need to continually incorporate more functional units. The sparse nature of the neural code in the DG is thought to be fundamental to dentate network function, yet the relationship between neurogenesis and low activity levels in the network remains largely unknown. Clues to the functional role of new neurons come from inquiries at the cellular as well as the behavioral level. Few studies have bridged the gap between these levels of inquiry by considering the role of young neurons within the complex dentate network during distinct stages of memory processing. We will review and discuss from a network perspective, the functional role of immature neurons and how their unique cellular properties can modulate the dentate network in memory guided behaviors.

The dentate gyrus (DG) hippocampal region is one of the most plastic regions in the mammalian brain, exemplified by its ability to generate adult-born principal neurons that integrate into the pre-existing network. Great effort has been made to understand the process and regulation of neurogenesis, and recently several groups have sought to determine the functional role of adult-born neurons in the DG. We postulate that in order to fully understand the functional role of adult-born neurons in the DG it will be necessary to consider the complexity of the local neural-network into which they integrate, focusing on network level mechanisms of DG computations and studying the contribution of adult-born neurons to those computations.

We will focus on one aspect of the DG that is critical to several theories of the role of the DG in memory processing: the observation that activity levels in the DG network are sparse. Both the proportion of active neurons and the action potential rates of active neurons are relatively low compared with other brain regions. Intuitively the observation of low activity levels introduces a puzzle; why would such a silent network require the constant addition of adult-born neurons? In other words, what is the relationship between adult-born neurons in the DG and the sparse encoding scheme implemented by the network? We will discuss two possibilities in the context of recent findings in the field, one, that adult-born neurons are themselves the small proportion of active cells in the DG at any given time and are thus “carrying the message,” or two, that adult-born neurons impose low activity levels in the DG by recruiting local inhibitory networks which act to suppress activity in mature DG granule cells, allowing a few to fire at any given time, thus “dictating the tone.” To gain insight into these possibilities we will review the unique properties of adult-born neurons in the context of the complexity of the greater DG network, focusing on linking proposed behavioral roles for adult-born neurons with long-standing theories of DG network coding. Throughout, we will highlight future experiments that could be done to properly bridge the gap between function and mechanism.

## The dentate gyrus is a sparse network

**Figure 1:**
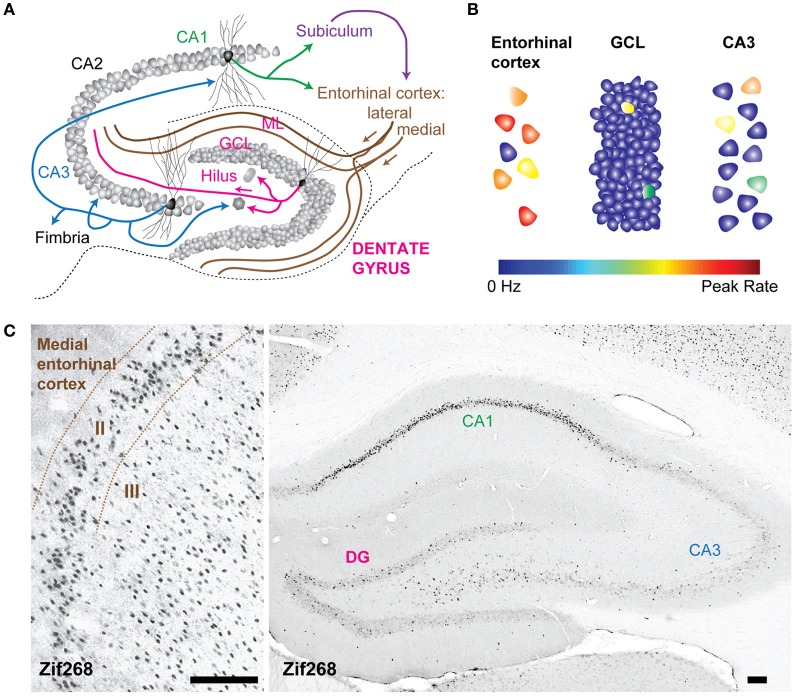
**The dentate gyrus is a sparse network. (A)** Schematic of the rat hippocampal circuit. Arrows represent excitatory axonal projections from representative principal cells within each layer to their downstream targets. For example, projections from dentate granule cells target hilar mossy cells and interneurons as well as CA3 pyramidal neurons. **(B)** Schematization of sparse activity levels in the dentate gyrus (DG) compared to the input layer (the entorhinal cortex) and the output layer (CA3). The color scale for each neuron indicates the firing rate from 0 Hz to the peak rate. Activity in the granule cell layer is low, because of a low proportion of neurons that fire action potentials and because the average firing rates of active neurons are low. **(C)** Expression of zif268, (activity dependent immediate early gene) in the rat entorhinal cortex (left) and hippocampus (right) after spatial exploration in an open field. Fewer neurons in the DG are positively labeled by zif268 immunoreactivity relative to the entorhinal cortex suggesting that a smaller proportion of the total population was active during spatial exploration. GCL, granule cell layer; ML, molecular layer. All calibration bars are 100 μm.

Classically, the DG is thought of as the first processing station of the hippocampal formation, comprising the first synapse of the “tri-synaptic pathway.” In simplified circuit diagrams, signals propagate from associative cortices, the lateral and medial entorhinal cortices, to the granule cells of the DG. From there signals are sent to downstream area CA3 and from CA3 to CA1. In reality, signals do not necessarily propagate in one direction along the tri-synaptic pathway, but instead ping-pong within sub-regions through associative pathways (Schwartzkroin et al., 1990), and even travel backwards through back projections from CA3 to the DG (Scharfman, 2007). Furthermore, there are direct connections from the entorhinal cortex to both CA3 and CA1 (Steward and Scoville, 1976; Witter and Amaral, 1991), bypassing upstream hippocampal processing (Figure 1A). Given that area CA3 receives the same direct input from the entorhinal cortices as does the DG, one might question what the additional role of the DG is in processing the same information and what additional contribution the DG makes to hippocampal-dependent memory. There may be instances when the DG transformed signal is more or less important for changing the activity patterns in the downstream CA3 recurrent network. To understand the relationship between the DG and its downstream targets, it may be helpful to identify the transformation of cortical signals performed by the DG.

Many lines of evidence converge to support the claim that the signal transformation performed by the DG is a “sparsification” operation because activity levels in the DG are lower than in the upstream cortical areas, and are thus a more sparse representation than the incoming neural activity pattern (Treves and Rolls, 1992; O'Reilly and McClelland, 1994; Acsady and Kali, 2007). The neural activity in the DG is sparse in two ways; both the proportion of active neurons as well as the mean firing rates of those neurons are low (Figure 1B). In the rat, there are approximately one million dentate granule cells (DGCs), which are the principal cells that send mossy fibers to CA3 (Boss et al., 1985; West et al., 1991). DGCs receive inputs from a smaller, highly active population of neurons in the entorhinal cortex (West et al., 1991), and the neural representation is thus considered to be expanded onto the larger number of DGCs (McNaughton and Morris, 1987). The large anatomical divergence can contribute to a sparse encoding scheme because even if several hundred neurons were simultaneously active, the proportion of active neurons would be low due to the large number of DGCs (Figure 1C). Indeed, the percentage of active DGCs in a given behavioral epoch has been estimated as corresponding to 1–2% of the total population, as indicated by labeling cells that express immediate early genes, such as c-Fos and Arc (Chawla et al., 2005; Tashiro et al., 2007; Alme et al., 2010). An increase in immediate early gene expression is thought to identify neurons that have recently undergone activity and has been used as a molecular tool to define active cell populations [However, it should be noted that neuronal activity observed in electrophysiological recordings is not always accompanied by an increase in immediate early gene expression, as indicated by the absence of elevated Arc expression in hippocampal neurons during rest or sleep when neuronal spiking is known to occur (Guzowski et al., 2006; Bramham et al., 2008; Miyashita et al., 2009)]. Furthermore, the firing rates of active neurons may also be described as sparse because neurons in the hippocampus have extremely low background rates of activity and are transiently activated under very specific conditions (Barnes et al., 1990; Jung and McNaughton, 1993; Leutgeb et al., 2007). Both aspects of sparse encoding are in stark contrast to the encoding scheme one synapse upstream in the entorhinal cortex, a network characterized by high levels of activity, both in the proportion of neurons active and in the mean firing rates of active neurons (Barnes et al., 1990). The position of the DG as the first processing station of the hippocampus, coupled with the sparse encoding scheme, led to the hypothesis that the DG translates cortical signals into a sparse code suitable for memory encoding (Treves and Rolls, 1994).

An aspect of the DG network that likely contributes to the sparse encoding scheme is the presence of a rich inhibitory network that interacts with the glutamatergic circuits supplying feedforward and feedback synaptic connections. The feedforward inhibitory drive onto DGCs is constant and strong, as several classes of hilar interneurons are more easily recruited by stimulation of the axons of the entorhinal cortex than are DGCs (Scharfman, 1991; Ewell and Jones, 2010). Strong feedforward inhibition would help mediate low mean firing rates in DGCs by ensuring that most signals arriving from the entorhinal cortex do not recruit spiking in DGCs, and when spiking does occur, it would be at lower rates because of the summation of large inhibitory potentials with excitatory potentials. Strong feedback inhibition also contributes to making the DG a competitive network, with a low proportion of active neurons in which activated DGCs excite interneurons that inhibit other DGCs (Rolls, 2010). Clearly, inhibition in the DG is a key aspect of the sparse coding scheme, therefore when searching to understand the role of adult-born DGCs in the DG; many clues will come from studying the interaction between adult-born neurons and interneurons.

Given the sparse spiking in DGCs, the DG network might only be effective by being coupled with an output mechanism by which DGCs can strongly excite their downstream targets without relying on mechanisms of input convergence; otherwise a sparse coding scheme might be counter-productive because no signals would be transferred. It has been demonstrated that single DGCs can reliably discharge interneurons and pyramidal CA3 cells (Henze et al., 2002), therefore, individual DGCs could “conditionally detonate” their post-synaptic targets in the CA3 network during the storage or recall of information during periods of elevated firing rate. Unlike any other cortical principal cell, DGCs have more than one terminal type along their axons. These include the large mossy terminals and two types of smaller terminals, filopodial extensions of the mossy terminals and *en passant* synaptic varicosities. The large mossy terminals make synaptic contacts with excitatory hilar mossy cells and pyramidal CA3 cells, whereas the filopodial extensions and the small *en passant* synaptic varicosities make synaptic contacts with GABAergic interneurons in the hilus and CA3 region (Acsady et al., 1998). What is the maturation time-line of these different synapse types in adult-born DGCs? Are there windows of time when adult-born DGCs would target only inhibitory circuitry or only CA3 pyramidal cells? A greater understanding of the differential targeting of local inhibitory circuits vs. output structures would have great implications for interpreting the role of adult-born DGCs to the output of the network and could shed light on the network mechanisms supporting dentate dependent memory.

The component of memory encoding that has long been theorized to be supported by the DG is pattern separation, which is thought to utilize a sparse coding scheme in the DG coupled with strong synaptic output to CA3 (Rolls, 1990; Treves and Rolls, 1994). Pattern separation is the process of transforming similar inputs into more dissimilar outputs, and is theorized to be necessary for reducing interference between similar memories in downstream area CA3 during memory encoding. Computational models of pattern separation predicted that similar experiences would be encoded by non-overlapping populations of neurons, and thus the DG would separate signals anatomically (O'Reilly and McClelland, 1994). However, experiments using electrophysiology in awake-behaving rodents have found that the same population of active DG neurons decorrelates subtle differences in sensory inputs, even if the first exposure to the environment was separated by several months (Leutgeb et al., 2007; Alme et al., 2010). The decorrelation can be accomplished by changes in firing rates and/or by changes in spatial firing patterns depending on the experimental manipulation, but always by modifying activity patterns within the same active neuronal population. In these studies the proportion of active cells and their mean firing rates were low; therefore the pattern separation operation utilized a sparse coding scheme, even though the mechanism deviated from modeled predictions.

The electrophysiological findings in awake-behaving animals give us a framework for the implementation of pattern separation, yet questions about the underlying mechanisms remain. The DG network is comprised of diverse excitatory neuron types, including mossy cells, immature, and mature DGCs (Neunuebel and Knierim, 2012), which cannot be distinguished using extracellular recordings *in vivo* because in most cases cell identity cannot be defined based on electrophysiological signature alone. Currently, neurons recorded using extracellular techniques can be segregated into broad classes, such as “principal neuron” and “interneuron” (Ranck, 1973; Wilson and McNaughton, 1993). Therefore, it is not clear whether the active cell population in the DG is composed of unique neuron subtypes and what each unique neuron population may contribute to dentate network computations that are critical for memory formation. Several recent computational models support the idea that pattern separation is mediated by the network as a whole, and that manipulations of any of the network components would affect the operation. For example, a model incorporating specific classes of hilar neurons shows that modulating the strength of hilar neurons may affect the ability of the dentate to perform pattern separation (Myers and Scharfman, 2009). Other computational models support the involvement of immature adult-born DGCs in the pattern separation computation (Aimone et al., 2011; Nogues et al., 2012).

An additional question arises from the fact that existing *in vivo* electrophysiological studies of pattern separation have been done exclusively in behavioral tasks in which animals are foraging in an open field with differing sensory features and different degrees of familiarity (Leutgeb et al., 2007; Alme et al., 2010), so it is not clear whether the insights to network mechanisms gained from these studies would apply to other dentate-dependent behavioral tasks. It is known that behavioral pattern separation tasks, in which animals must discriminate between adjacent spatial locations, are dependent on the DG (Gilbert et al., 2001; Morris et al., 2012; Kesner, 2013), however those studies did not focus on the contribution of individual neuron types. Several groups have recently found that manipulations of neurogenesis affect learning of contextual and spatial discrimination tasks, supporting a role for adult-born DGCs in behavioral pattern separation (Clelland et al., 2009; Creer et al., 2010; Sahay et al., 2011a; Kheirbek et al., 2012; Nakashiba et al., 2012). Thus, as a field we have some knowledge of the network mechanisms underlying pattern separation for one type of behavioral task, and evidence suggesting that adult-born DGCs are important for dentate computations in other types of behavioral tasks. Rather than dwelling on the gap in our knowledge, we think it is more productive to assume, until we have reason not to, that the network mechanisms would be similar, in that they may both rely on a sparse coding scheme, a hallmark of DG network coding. With that assumption in mind, do the results from behavioral studies imply that adult-born neurons are performing the computation underlying pattern separation, or do they imply that they are one of the many contributors to a well orchestrated neural-network computation? One way to differentiate those possibilities is to examine the relationship of adult-born DGCs to sparse coding in the dentate, thus focusing the inquiry on the mechanisms underlying function. Do adult-born DGCs contribute to a sparse dentate representation by serving as the active cell population recruited to encode distinct events (carrying the message) or do adult-born DGCs interact with the local DG network to sculpt the patterns of activity and establish sparse coding within a heterogeneous active cell population (dictating the tone). Clues for understanding the relationship between adult-born DGCs and sparse coding in the DG come from experimental studies aimed to determine the maturation of adult-born DGCs overtime, and how they connect with components of the dentate network.

## The dentate gyrus is a neurogenic network

**Figure 2:**
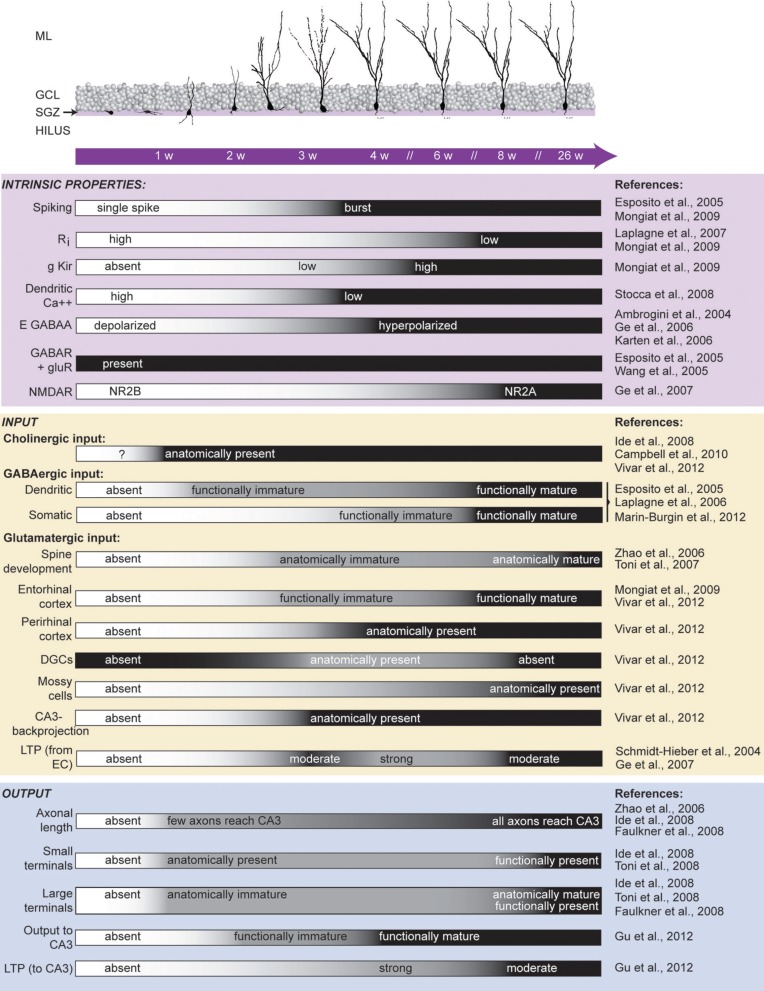
**Maturation of adult-born DGCs.** Critical time points over a period of several weeks are indicated within the purple bar. **(Top)** The morphological maturation of dendritic arbors of adult-born DGCs (adapted from Esposito et al., 2005). Axonal arbor development is not shown. **(Bottom)** Summary of reported findings during the maturation of adult-born DGCs. Each characteristic is listed to the left with references from which the timeline is depicted cited to the right. Black indicates the developmental age when the defined characteristic is indistinguishable between immature and mature DGCs (see primary literature for the specific time points that were included in each experiment). Ri, input resistance; g Kir, inward rectifier potassium conductance; DGCs, dentate granule cells; LTP, long-term potentiation; EC, entorhinal cortex. ML, molecular layer; GCL, granule cell layer; SGZ, sub-granular zone.

The DG is one of few unique adult brain regions where functional units are continuously generated and incorporated into the pre-existing network (Kaplan and Hinds, 1977; Kuhn et al., 1996; Eriksson et al., 1998). Neural progenitor cells in the dentate sub-granular zone have the ability to generate principal dentate neurons in addition to glial cells (Gage, 2000; Van Praag et al., 2002). The maturation of adult-born DGCs is multi-faceted (Figure 2), and many aspects have been reviewed elsewhere (Piatti et al., 2006; Zhao et al., 2008; Mongiat and Schinder, 2011; Kim et al., 2012; Song et al., 2012). We would like to focus on a time-point when adult-born DGCs have achieved a stage of maturation when they could have a functional influence on the DG network dynamics but are still distinct from mature DGCs. The neuronal age of adult-born DGCs capable of influencing the network would coincide with a stage of development when they have dendrites receiving inputs from cortical and local sources, they have the ability to release neurotransmitter, and they have axons that contact post-synaptic targets. We consider these attributes to be the basic requirements for a functional unit to be capable of performing an input-output transformation, and thus impacting the network in a meaningful way. At approximately 4 weeks in the maturation timeline, adult-born DGCs meet these criteria, and importantly from 4 to 8 weeks several neuronal properties are still emerging, thus distinguishing these immature adult-born DGCs from the population of mature DGCs. Specifically, at 4 weeks, adult-born DGCs have intrinsic properties that confer hyperexcitability, have enhanced plasticity at both their input synapses from the entorhinal cortex and at their output synapses to CA3, and receive input directly from mature DGCs, all of which distinguish them from the population of mature DGCs.

Immature neurons are highly excitable; they display high input resistance, low inward rectifier potassium conductance, and a low threshold for Ca^++^ spikes (Schmidt-Hieber et al., 2004; Mongiat et al., 2009). In addition to their intrinsic excitability, immature DGCs also have weaker perisomatic inhibition with slower kinetics than mature neurons (Marin-Burgin et al., 2012), allowing them to be more sensitive to their excitatory inputs. Moreover, their excitatory inputs from medial entorhinal cortex can be potentiated during the critical period when DGCs are 4–6 weeks old, which is dependent on NR2B-containing N-methyl-D-asparate (NMDA) receptors (Ge et al., 2007). Plasticity mediated by NR2B- containing NMDA receptors in adult born DGCs seems functionally relevant because discrimination learning of two highly similar contexts is dependent on NR2B-mediated plasticity, suggesting that manipulating only the input strength to immature DGCs is sufficient to disrupt DG encoding (Kheirbek et al., 2012). Furthermore, it is possible that most long-term potentiation (LTP) occurring in synapses between the medial entorhinal cortex and DGCs is selective to adult-born DGCs because LTP there can be entirely blocked by only removing neurogenesis by γ-irradiation (Snyder et al., 2001). However, the initial impairment in LTP can be rescued over time, suggesting other components of the network can eventually compensate (Singer et al., 2011). Together, these studies suggest that adult-born DGCs are highly excitable within a critical period, both because of their intrinsic properties and because of their local circuit interactions.

## Could hyperexcitable, immature adult-born DGCs be the sparse active cells of the DG network?

In the adult DG network, 3% of DGCs are newborn neurons, which integrate into the pre-existing circuit (Cameron and McKay, 2001; Dayer et al., 2003). At first glance, we may think that 3% of DGCs would be insignificant in a network of millions of neurons, in which only 2% of neurons are active during behavior. However, given that immature neurons have unique cellular properties such as enhanced excitability, it seems plausible that the sparse activation of DGCs which is critical for dentate dependent memory processing, could be achieved by only activating the immature DGCs.

Whether the enhanced excitability in immature DGCs biases them toward being the only active principle neurons of the dentate network is controversial. As expected from the disparity in excitability between immature and mature DGCs, it was shown *in vitro* with calcium imaging that afferent stimulation activates a higher number of 4-week old DGCs compared to mature DGCs. Furthermore, 4-week old DGCs require less input strength to reach action potential threshold compared to mature DGCs (Marin-Burgin et al., 2012). These data suggest that 4-week old DGCs could be more likely to be active *in vivo*. However, it is clear that immature DGCs are not the only active neurons in the dentate network because mature DGCs express cFos after training and memory recall in spatial memory and in contextual fear memory, regardless of whether they were born in the embryonic, post-natal, or adult phase of development (Stone et al., 2011). Still, it would be interesting to know whether 4-week old DGCs are preferentially recruited *in vivo* as would be predicted from *in vitro* studies. Perhaps immature DGCs could be the active members of the DG in particular situations; yet in others they could be functionally equivalent to older DGCs. Indeed, Trouche et al. (2009) have demonstrated that the recruitment of 5-week old immature DGCs was situation-specific.

The debate whether immature DGCs are the active members of the network naturally leads to the question of whether they have functional connectivity with downstream CA3. By 4 weeks of age DGCs have large mossy terminals (>3 um) in CA3, however, they still appear structurally immature because they have fewer synaptic vesicles and active zones, and contact fewer CA3 spines compared to mature DGCs (Faulkner et al., 2008; Toni et al., 2008). Despite structural immaturity, excitatory postsynaptic and monosynaptic currents have been recorded *in vitro* on CA3 pyramidal cells after optical stimulation of adult-born DGCs as young as 2 weeks old. By 4 weeks the output of adult-born DGCs matches the responses of mature DGCs (Gu et al., 2012). Moreover, Gu et al. (2012) have gone one step further and demonstrated that optical stimulation of immature DGCs (3–4 weeks old) but not mature (8 weeks old) DGCs induced LTP of the excitatory field potential of area CA3 in anesthetized mice. This work eloquently shows that immature DGCs are functionally connected to CA3 and thus would be capable of “carrying the message,” however the impact that immature DGCs would have on CA3 would also depend on their recruitment of feedforward inhibitory interneurons. Indeed, 4-week old DGCs may have a net inhibitory effect in CA3 as Restivo et al. (2012) have recently found that 4-week old DGCs have significantly more filopodia stemming from the large mossy fiber terminals compared to the mature DGCs. These filopodial extensions selectively innervate GABAergic cells (Acsady et al., 1998; Ruediger et al., 2011). Therefore, it would be critical to study the impact of 4-week old DGCs on the excitation/inhibition balance in CA3, by comparing the relative activation of pyramidal cells versus interneurons by 4-week old DGCs, or conversely, by silencing 4-week old DGCs and determining whether CA3 is more or less excitable.

The DG recruitment of GABAergic interneurons in the CA3 field may be a crucial component to proper memory encoding. A recent study has demonstrated that, during learning, structural changes of synapses from DGCs to CA3 interneurons determines memory precision (Ruediger et al., 2011), supporting the idea that feedforward inhibition from the DG to CA3 may be mechanistically relevant for aiding in separating representations in CA3 during memory encoding. Although these data suggest that the DG-recruited excitation/inhibition balance in CA3 is behaviorally relevant during memory encoding, they do not distinguish a role for adult-born versus developmentally born DGCs. If immature adult-born DGCs were the active members of the DG population during learning, then it would be expected that the output from adult-born DGCs to interneurons in CA3 would undergo learning dependent changes. In support of that prediction, Restivo et al. (2012) found a learning-dependent increase of filopodia terminals on adult-born DGC axons and a positive correlation between the neuronal activity of immature DGCs and CA3 interneurons, assayed with elevated cFos expression. Therefore, immature adult-born DGCs could facilitate learning and memory precision due to their ability to plastically target CA3 interneurons and modulate inhibitory tone.

An important role for immature DGCs as the active population of the DG network is also suggested by one recent study that has silenced the activity of all DGCs except immature DGCs and shown that dentate dependent memory is facilitated. Nakashiba et al. (2012), using genetic tools, created a triple transgenic mouse in which DGCs expressed tetanus toxin (TeTX) in an inducible manner mediated by a Tet-OFF, CRE-loxP recombination system. TeTX cleaves the synaptic vesicle protein synaptobrevin abolishing neurotransmitter release and synaptic neuronal transmission in the output synapses. The investigators took advantage of the fact that young adult-born DGCs are unaffected by their manipulation because Cre-loxP recombination occurs only in neurons that are approximately 2 weeks old, and then takes additional time to confer functional expression. Thus, because of the time lag, the output synapses of most adult-born DGCs younger than 4–6 weeks remain functional, while the output synapses of DGCs older than 6 weeks are silenced. Using this technique, they were able to silence the majority of mature DGCs, while leaving intact the neuronal transmission of the small population of immature DGCs. Under these conditions, the authors found a facilitation of contextual fear discrimination learning of highly similar contexts. Moreover, they showed that the facilitation in learning was dependent on immature DGCs because it was abolished by blocking neurogenesis with X-ray irradiation, regardless of whether the older DGCs were silent or active. It is interesting to consider that in their primary result, the investigators experimentally imposed sparseness on the DG by silencing 98% of the population (the mature DGCs). Is it possible that imposing that sparseness is what caused the facilitation in learning rather than special attributes inherent to the age of the remaining DGC population? One way to test this possibility is to determine whether the same facilitation in learning occurs after imposing the same degree of sparsity within the dentate network, but with the remaining population composed entirely of mature DGCs.

## Could immature adult-born DGCs impose the sparseness on the DG network?

The most critical component that determines the sparseness of the DG network is the level of inhibition. DGCs modulate inhibition in the DG network through direct feedback and lateral inhibition (Freund and Buzsaki, 1996) and, indirectly, by innervating the excitatory hilar mossy cells that target interneurons locally and DGCs in distant lamella (Scharfman, 1995). Therefore, knowledge of when, how, and to which neuronal targets adult-born DGCs functionally connect is essential to understanding their role in the network. In the hilus, structural analysis showed that immature and mature DGCs had mostly mossy fiber boutons of small size, suggesting that most of their targets are GABAergic interneurons (Acsady et al., 1998; Ide et al., 2008; Toni et al., 2008). Furthermore, when adult neurogenesis was absent for 10 weeks, the inhibitory innervation in the DG was decreased, suggesting that adult-born DGCs influence the local balance of excitation and inhibition in the DG network (Singer et al., 2011). To definitively understand the relationship between adult born DGCs and interneurons, studies of functional connectivity need to be done. Toni et al. (2008) expressed channel rhodopsin in adult generated DGCs, allowing them to optically stimulate only adult born DGCs and record post-synaptic responses in various interneuron types in the DG network. They found functional connectivity between adult-born DGCs and putative GABAergic interneurons of several classes as well as with hilar mossy cells. Unfortunately, because of technical limitations, they were not able to reliably identify connections of adult-born DGCs that were younger than 15 weeks. However, with techniques that make it possible to optically stimulate adult-born DGCs as young as 2 weeks in age (Gu et al., 2012), one could imagine studying the connectivity with the multitude of possible post-synaptic targets, at various time points in their maturation. It is likely that adult-born DGCs would have different effects on the local network at different ages, possibly providing flexibility to dentate computations such that by regulating the number of adult-born DGCs of a certain age, the computation performed by the network would be fundamentally different.

Even without the characterization of the functional connectivity with various targets of the local network, many groups have theorized that the role of adult-born neurons is to modulate the neuronal activity of the larger population of mature DGCs (Ming and Song, 2011; Sahay et al., 2011b). Supporting this idea, Lacefield et al. (2012) have demonstrated that when neurogenesis was abolished, such that the remaining adult-born DGCs were at least 6 weeks or older at the time of recording, DG network oscillations in anesthetized mice were impacted. Under these conditions, there was a marked increase in the amplitude of spontaneous gamma frequency bursts, which were shown to be dependent on the input from the entorhinal cortex. Interestingly, the action potentials recorded from neurons in the dentate became synchronized during gamma bursts when neurogenesis was absent, even though the percentage of action potentials that occurred within gamma bursts did not change. Increased synchrony and tighter phase locking were also observed during periods of theta oscillations. Confirmation of these data in awake-behaving animals is necessary, however, because the mechanisms that generate network oscillations in awake animals may be different than under anesthesia (Ylinen et al., 1995a,b). It has been previously shown that spike timing in principal neurons is precisely regulated by fast-spiking interneurons (Pouille and Scanziani, 2001), the same interneurons essential for coordinated gamma oscillations (Korotkova et al., 2010). Therefore, it seems likely that immature DGCs have some impact on local inhibitory networks, such that when neurogenesis is removed, the oscillatory dynamics are altered. Given the complexity of the local microcircuit it is difficult to predict how immature DGCs may be mediating these local effects, again highlighting the need for identifying their functional connections with various components of the network, both *in vitro* and in awake-behaving animals.

Neural synchrony is thought to be important for working memory, in which recently acquired information is held “on-line” for sustained periods of time (Durstewitz et al., 2000). Several groups have postulated a role for gamma oscillations in working memory tasks (Jensen et al., 2007; Lisman, 2010), suggesting that manipulations of gamma oscillations might affect working memory performance. Indeed, when local network processing was altered by reducing gamma oscillations in mice by knocking down NR1 in hippocampal parvalbumin-positive interneurons, there was a deficit in spatial working memory (Korotkova et al., 2010). On the other hand, under anesthesia, gamma oscillations increase when neurogenesis is blocked (Lacefield et al., 2012). It would therefore be expected that adult-born DGCs would negatively impact spatial working memory. Indeed, ablation of neurogenesis for 3 months improves spatial working memory performance, but only for difficult versions of the task (Saxe et al., 2007). The improved performance when neurogenesis was blocked could be a result of increased gamma oscillations, but recording studies of local field potentials in animals without neurogenesis performing working memory tasks would need to be done and compared to animals with neurogenesis intact in order to confirm this interpretation.

If immature DGCs were recruiting feedback and lateral inhibitory circuits in the DG, we would expect that blocking neurogenesis may release inhibition and result in increased numbers of active DGCs. Burghardt et al. (2012) found a specific up-regulation of Arc expression in the dentate granule cell layer of mice lacking neurogenesis. This increase in the active number of DGCs occurred specifically after the neurogenesis deficient mice experienced a conflicting experience, namely to actively avoid a novel location of a shock zone in the same context where they had previously learned a shock zone in a different spatial location. This behavior was found to be dentate dependent and mice without neurogenesis exhibited a performance deficit in comparison to controls. Their findings, along with other recent studies, showed that immature DGCs are necessary to reduce interference when learning a behavioral task that is contextually similar to a task that was learned previously, but not when the two similar conflicting tasks were learned simultaneously (Burghardt et al., 2012; Luu et al., 2012; Tronel et al., 2012; Winocur et al., 2012). It is possible that interference occurs under the condition of learning at different times because immature DGCs modulate the balance between encoding and retrieval mechanisms in the downstream CA3 network. Here neurogenesis may be necessary for the CA3 network to learn a new rule or shock location. However, it is also possible that the immature neurons act to suppress background noise through feedback or feedforward inhibition, sparsifying the representation, thus reducing overlap in the active population. More studies assaying activity patterns in the DG under conditions when neurogenesis is altered and behavior is impaired need to be done to distinguish between these possibilities.

## Conclusions and future advances

**Figure 3:**
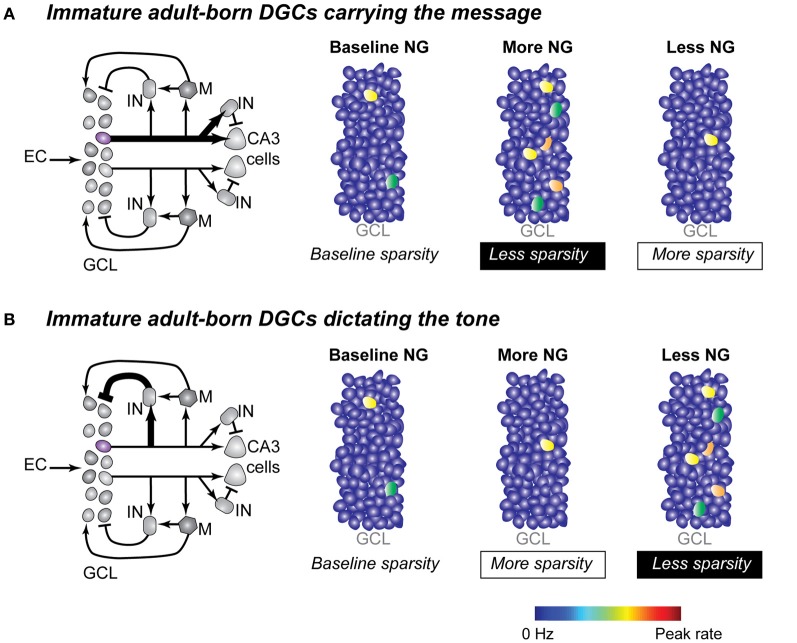
**Two possible mechanisms for adult-born DGCs to determine sparsity in the dentate network. (A)** Immature DGCs act as the active neuronal population in the DG network. Left panel: simplified local dentate circuit in which the output of one immature DGC (purple) and the output of one mature DGC (gray) are represented. Arrows and bars represent excitatory and inhibitory synapses respectively. Line weight represents signal strength with thicker lines suggesting stronger output strength, through either synaptic strength and/or number of synaptic connections. Right panels: changes in dentate network sparsity as a result of increasing or decreasing neurogenesis under conditions when immature neurons carried the primary output of the DG (symbols as described in Figure 1B). **(B)** Immature DGCs act as modulators of the inhibitory tone in the dentate network. Left panel: simplified dentate circuit in which immature DGCs more strongly influence the local interneuron population. Right panels: changes in dentate network sparsity as a result of increasing and decreasing neurogenesis under conditions when immature neurons modulate the inhibitory drive on other DGCs. EC, entorhinal cortex; GCL, granule cell layer; IN, inhibitory interneuron; M, mossy cell; NG, neurogenesis.

Currently in the field of neurogenesis, there is ample data describing the cellular properties of adult-born neurons and recently there has been a fast-paced accumulation of data describing the functional role of adult-born neurons in memory behaviors. The gap in our knowledge is studies that integrate these two lines of study and that illuminate the functional role of adult-born DGCs at the network level. In this review we have focused on two possible roles that active adult-born DGCs might play in DG network computations, and we specifically considered the relationship of neurogenesis to sparse network coding. Immature DGCs could carry the message directly to the downstream CA3 region by being the sparse active members. Alternatively, they could impose the tone in the DG network through interactions with interneurons in the local circuit that mediate the maintenance of the sparse coding scheme and the selection of the appropriate DGCs, immature and mature, to carry the message (Sahay et al., 2011b). Although there may be more possible roles for adult-born DGCs in the DG network, focusing on these two simple cases yields straightforward and testable implications for network activity. If immature adult-born neurons were the sparse active members in the dentate network, then it would be expected that blocking neurogenesis would lead to fewer active neurons in the dentate network and to more active neurons when neurogenesis is accelerated. An increase in active cell numbers, resulting in a less sparse network, would have potential ramifications for the ability of the network to perform pattern separation operations (Figure 3A). If instead immature DGCs were enforcing the sparse activation of the entire dentate network through feedback and lateral inhibition, then it would be expected that blocking neurogenesis would lead to a larger active neuronal ensemble and a network with decreased sparsity. In this case acceleration of neurogenesis would result in amplified sparsity and presumably a network better primed to perform pattern separation (Figure 3B).

Neurogenesis is an extraordinary plastic phenomenon that offers adaptive advantages to the DG due to the fact that it can be modulated by behavior and experience. Changes to the local dentate network as a result of experience can modulate the generation, survival, rate of maturation, and integration of adult-born DGCs (Piatti et al., 2006, 2011; Tashiro et al., 2006; Ma et al., 2009). Specifically, it has been shown that the dentate network can sculpt the generation, maturation and survival of different cohorts of DGCs according to behavioral task demands and according to distinct physiological states (e.g., exercise and stress) (Warner-Schmidt and Duman, 2006; Dupret et al., 2007; Inokuchi, 2011; Xu et al., 2011; Marin-Burgin and Schinder, 2012), which permits the DG to optimize network computations that are modulated by neurogenesis. A neural network primed to perform distinct computations may alter the contribution of the DG to memory processing (Inokuchi, 2011; Marin-Burgin and Schinder, 2012). Importantly, the modulation could differ for different types of memory if distinct activity patterns from divergent inputs (i.e., amygdala and entorhinal cortex) differentially engage immature DGCs, modifying their roles in the local dentate circuit in response to the nature of converging input patterns. For example, the two major divisions of the entorhinal cortex, the medial and lateral entorhinal cortices, are thought to carry distinct types of information (Burwell, 2000; Manns and Eichenbaum, 2006; Kerr et al., 2007) and have been shown to synapse onto anatomically segregated portions of DGC dendrites (Hjorth-Simonsen, 1972; Hjorth-Simonsen and Jeune, 1972; Witter et al., 1989; Witter, 2007). An inverse response of immature DGCs to the activation of distal or proximal dendritic inputs in comparison to mature neurons would bias their contribution to the transformation of distinct types of representations critical for memory formation. Hence the modulation of the number of new neurons in the circuit, would serve to selectively alter the response of the dentate neural network to the same input patterns over time. It is intriguing to imagine how recent experience could modulate neurogenesis, thus influencing the coding scheme in the dentate for sustained periods of time and consequently memory processing. Neurogenesis lends itself as the perfect candidate to offer the DG the flexibility to employ different coding schemes for different periods in one's life. Recordings during various types of behavioral tasks under conditions where neurogenesis has been increased or decreased would be necessary to fill the gap between our understanding of the behavioral role of immature DGCs and our understanding of the network mechanisms supporting diverse behaviors. Such studies will simultaneously reveal answers to pressing questions about neurogenesis and continue to reveal broader insights into DG and hippocampal function.

### Conflict of interest statement

The authors declare that the research was conducted in the absence of any commercial or financial relationships that could be construed as a potential conflict of interest.
